# Aquatic Exercise in Physical Therapy Treatment for Fibromyalgia: Systematic Review

**DOI:** 10.3390/healthcare12060701

**Published:** 2024-03-21

**Authors:** Manuel Rodríguez-Huguet, Carmen Ayala-Martínez, Pablo Góngora-Rodríguez, Miguel Ángel Rosety-Rodríguez, Rocío Martín-Valero, Jorge Góngora-Rodríguez

**Affiliations:** 1 Department of Nursing and Physiotherapy, University of Cádiz, 11009 Cádiz, Spain; manuel.rodriguez@uca.es (M.R.-H.); jorge.gongora@uca.es (J.G.-R.); 2 Doctoral School, University of Cádiz, 11003 Cádiz, Spain; pablo.gongorarodriguez@alum.uca.es; 3 Move-It Research Group, Biomedical Research and Innovation Institute of Cadiz, Puerta del Mar University Hospital, University of Cádiz, Plaza Fragela, s/n, 11003 Cádiz, Spain; miguelangel.rosety@uca.es; 4 Department of Physiotherapy, Faculty of Health Sciences, University of Málaga, 29071 Málaga, Spain; rovalemas@uma.es

**Keywords:** aquatic exercise, fibromyalgia, physical therapy

## Abstract

Fibromyalgia is a chronic condition characterized by musculoskeletal pain. The aim of this study was to synthesize scientific evidence on the effects of aquatic exercise programs on pain and quality of life in individuals with fibromyalgia. This review was carried out using the following databases in January 2024: Cochrane Library, PEDro, PubMed, SCOPUS, and Web of Science. Four clinical trials focusing on aquatic exercise as a treatment for patients with fibromyalgia were selected. These trials were published in English between 2019 and 2024. Pain recorded using the Visual Analog Scale (VAS) and quality of life with the Short Form-36 Health Survey (SF-36) or Fibromyalgia Impact on Quality of Life (FIQ) were the most commonly analyzed variables. This review was carried out according to the PRISMA statement and was registered in PROSPERO (CRD42024510219). The results in terms of pain and quality of life were positive. In conclusion, these findings support the incorporation of aquatic exercise into fibromyalgia physical therapy treatment. However, the benefits could be equivalent to those of other exercise modalities, underscoring the need for individualized adaptation to each patient’s needs.

## 1. Introduction

Fibromyalgia is a chronic condition characterized by diffuse musculoskeletal pain, fatigue, sleep disturbances, and other cognitive and somatic symptoms such as hyperalgesia at specific tender points and headaches [1,2]. The symptomatology limits the functional independence of patients and diminishes their quality of life. It is a syndrome that affects between 0.2% and 6.6% of the global population, particularly women over the age of 50 [2,3]. The pathophysiology of this disorder is not clearly defined [2,4], which complicates diagnosis and treatment, although its origin could be related to a combination of genetic, biological, psychological, and environmental factors [5,6,7,8].

Treatment for fibromyalgia typically involves a multidisciplinary approach, including a combination of medications, physical therapies, stress management techniques, and lifestyle changes. The chronic nature of the disease and its significant impact on patients’ lives result in high socioeconomic costs associated with this condition [9]. This underscores the necessity for further research to be conducted in order to provide effective treatments, taking into account the need for treatment to be individualized and adapted to the patient’s capabilities [10].

Active exercise plays a pivotal role in the physiotherapeutic treatment of fibromyalgia [11]. Though challenging for those afflicted with this condition, incorporating a regular, supervised exercise program can yield significant benefits in symptom management. Specific exercises targeting strength, flexibility, and aerobic capacity not only aid in improving overall physical condition, but also contribute to reducing pain, enhancing sleep quality, and boosting emotional well-being in fibromyalgia patients [11,12,13]. Moreover, active exercise can play a crucial role in promoting autonomy and enhancing the quality of life for those affected by this condition, equipping them with practical tools to effectively manage their condition in the long term [14]. Exercise in an aquatic environment has been proposed as an option within the active exercise program, and its specific effects must be assessed [11,15]. The aquatic environment is a low-impact adapted environment that could facilitate joint mobilization and exercise and increase patient motivation.

The objective of this review was to synthesize scientific evidence on the effects of aquatic exercise programs on pain and quality of life in individuals with fibromyalgia, assessing their potential for inclusion in physical therapy treatment programs.

## 2. Materials and Methods

### 2.1. Review Protocol and Search Strategy

The search for this systematic review was conducted following the Preferred Reporting for Systematic Reviews and Meta-Analyses (PRISMA) guidelines and the review was registered with the International Prospective Register of Systematic Reviews (PROSPERO) with code: CRD42024510219. The search for articles was conducted in January 2024 by two reviewers, consulting the main health sciences databases. Specifically, the following databases were used: Cochrane Library, PEDro, PubMed, SCOPUS and Web of Science. The PICOS model was applied to establish the research question: P = population, fibromyalgia patients; I = intervention, aquatic exercise; C = comparison, other physical therapy treatments; O = outcomes, pain and quality of life; S = study design, randomized clinical trials.

To enable the search in the cited databases, the DeCS/MeSH platform was initially used to define the appropriate descriptors, which were: “aquatic exercise” and “fibromyalgia”. These terms were related to the Boolean operator “AND”, so the search in the different databases began with the following structured language: “aquatic exercise AND fibromyalgia” because this formula allows you to reproduce and update the search.

### 2.2. Eligibility Criteria, Study Selection and Data Collection Process

This review applied specific selection criteria, targeting randomized controlled trials (RCTs) published in the last five years (2019 to 2024), available in English or Spanish. The studies considered were focused on individuals diagnosed with fibromyalgia who underwent physical therapy treatment that incorporated active exercises performed in an aquatic environment. Once duplicate references were removed and the specified filters were applied, the selection process proceeded to the screening of articles based on title and abstract readings. The screening was performed by two independent reviewers (J.G.-R. and C.A.-M.), and in cases of conflict a third reviewer (M.R.-H.) from the research team was consulted. The reviewers verified that the articles met the previously established selection criteria. Additionally, it was required that the articles include pain and quality of life among their primary variables.

To ensure methodological qualities across studies, a minimum threshold of 7 points on the PEDro scale was imposed as a criterion. The score of all selected articles was checked on the PEDro website. The intention was to collect the most current studies with high methodological quality. The entire sample, along with allocation within each group, was examined in all studies. Detailed records covering variables, treatment modalities, intervention durations, follow-up intervals, and trial results were meticulously compiled to facilitate comprehensive scrutiny and analysis. However, a qualitative analysis was chosen due to the heterogeneity of the treatment proposals included.

### 2.3. Quality Assessment of Studies

The methodological quality of scientific articles is an essential element to assess the validity and reliability of the findings and thus determine the inclusion of the articles in the review. The PEDro scale (Physiotherapy Evidence Database) is a tool widely used for this purpose in the field of physiotherapy. This scale is made up of 11 items and evaluates aspects such as allocation concealment, blinding of participants and evaluators, and follow-up of lost patients, among others [16]. Despite having 11 questions to evaluate, the final score of each article is calculated ignoring the first item, obtaining a score from 0 to 10. For this review, only articles with a minimum score of 7 were selected.

Furthermore, thanks to the PEDro scale, it is possible to monitor the risks of bias. It encompasses factors that facilitate the assessment of risk of bias. Specifically, items 2 and 3 address selection bias, items 5 and 6 pertain to performance bias, and item 7 focuses on detection bias. Consequently, a high PEDro score signifies strong methodological integrity and minimal risk of bias, while a low score suggests an increased risk of bias and compromised methodological quality.

## 3. Results

### 3.1. Study Selection

**Figure 1:**
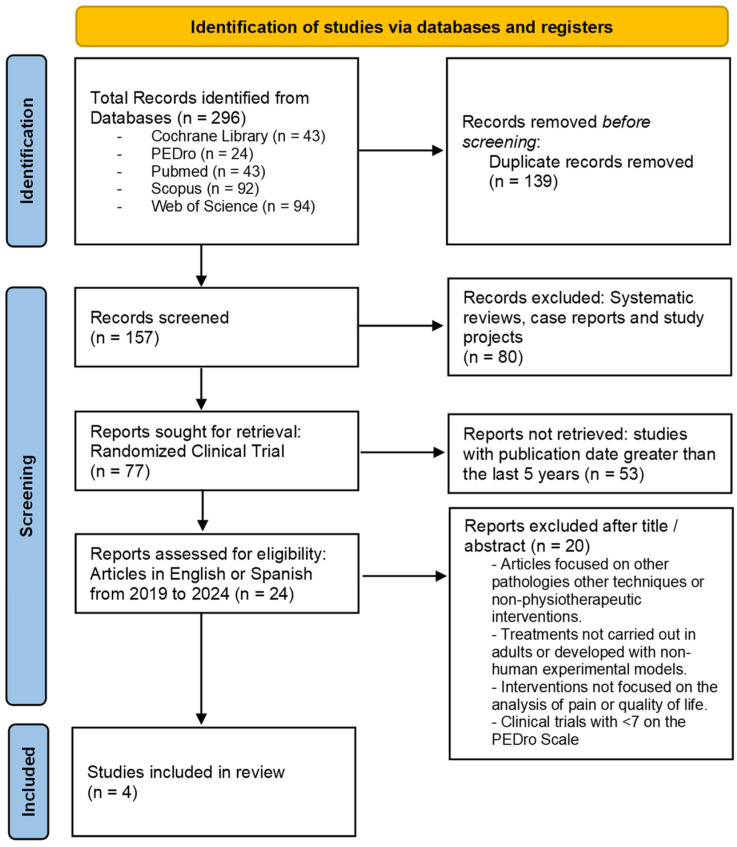
PRISMA flow diagram. Identification of the results obtained from the databases.

The PRISMA flow diagram (Figure 1) shows the screening process and the systematic review phases according to the established selection criteria. The initial search offered a total of 296 studies, of which 4 met all of the requirements and were finally selected.

The total number of results analyzed was 157 articles, after eliminating duplicate results. The type of article and the language in which they were available reduced the number of articles to 24. Finally, 20 articles were excluded due to their score on the PEDro scale or because they were dedicated to other types of interventions not based on aquatic exercise or studied other pathologies.

### 3.2. Sample Population Characteristics and Methodological Quality Assessment

In total, the combined sample size across the selected studies comprised 157 subjects, with an average age of 48.53 years [17,18,19,20]. All participants were women; in the majority of selected studies, female sex was established as an inclusion criterion [17,19,20], and even when the recruitment was aimed at both sexes, the sample obtained was also entirely women [18]. The selection of the sample was determined by a diagnosis of fibromyalgia syndrome, following the diagnostic guidelines of the American College of Rheumatology; individuals with another associated disease were excluded [17,18,19,20].

Table 1 shows the most relevant information of each of the selected articles, such as the number of participants and the distribution of the groups, the clinical variables of the study, and the follow-up time of the interventions. Table 2 includes information about the aquatic exercise treatment, alternative interventions, and the most outstanding results of each investigation.

The detailed characteristics of each intervention and the statistical results of each study are indicated in the following sections.

Table 3 represents the total score of each investigation on the PEDro scale and its individual assessment for each item. In this way, the methodological quality and the possible risk of bias have been assessed, obtaining a score of 8 in all of the articles analyzed [17,18,19,20]. Therefore, the consistency of the clinical trials studied was appropriate, and the risk of bias can be considered low. The main deficiency is observed in the blinding of patients and therapists, which is especially complicated in this type of intervention.

### 3.3. Outcomes, Measurements, and Assessment Time

The analyzed articles recorded changes in the clinical manifestations of fibromyalgia syndrome and other associated study variables. The assessment of pain through the Visual Analog Scale (VAS) scale was the most common study variable, appearing in all of the selected results [17,18,19,20]; likewise, Salm et al. (2019) [20] also included pain monitoring using the Short Form McGill Pain Questionnaire (SF-MPQ). Furthermore, the research by Andrade et al. (2019) [17] used the VAS to estimate fatigue and well-being.

Along the same lines, the assessment of health-related quality of life and the impact of fibromyalgia on the quality of life were widely studied. Thus, the Short Form-36 Health Survey (SF-36) was included in two of the clinical trials [17,19], and the Fibromyalgia Impact on Quality of Life (FIQ) questionnaire was used in all studies [17,18,19,20].

It is possible to highlight that De Medeiros et al. (2020) [19] valued beliefs, fear, and catastrophism with the Fear Avoidance Beliefs Questionnaire (FABQ) and Pain-Related Catastrophizing Thoughts Scale (PRCTS), as well as sleep quality with the Pittsburgh Sleep Quality Index (PSQI), which was also included in another article [17]. On the other hand, Britto et al. (2020) [18] included an assessment of the digital palpation of tender points according to standardized guidelines and also valued flexibility with Well’s bench sit and reach test. Salm et al. (2019) [20] incorporated infrared thermography analysis and studied biochemical markers with the analysis of the serum level of cytokines. In addition, Andrade et al. (2019) [17] assessed the Pressure Pain Threshold (PPT), Beck’s Anxiety Inventory (BAI), Beck’s Depression Inventory (BDI), and other variables related to intensity and response to exercise, such as oxygen uptake (VO_2_) and the submaximal cardiopulmonary exercise test (CPET).

Regarding the follow-up time of the interventions, three of the selected studies exclusively carry out a baseline and post-treatment evaluation [18,19,20], and one of the investigations used a longer-term follow-up, with an evaluation 32 weeks after the start of the study and 16 weeks after the end of treatment [17].

### 3.4. Interventions, Protocols and Effects of Treatments

The treatment proposals analyzed coincide in including individualized interventions or sessions in small groups under the supervision of a physiotherapist specialized in aquatic exercise [17,18,19,20]. The intervention protocols of Britto et al. (2020) [18] and De Medeiros et al. (2020) [19] included a comparison of an aquatic exercise treatment versus another exercise modality on land, such as Pilates exercises on the floor [19] or an exercise program (warm-up, active stretching, strengthening, and relaxation) on land [18]. However, Salm et al. (2019) [20] included aquatic therapy in both groups. In that case, active exercise was accompanied by a temperature-raising therapeutic procedure in one group and a placebo T-shirt therapy in the other.

Moreover, Andrade et al. (2019) [17] described the full design of the protocol in a previous publication [21], and included a control group instructed to maintain their baseline levels of physical activity. With respect to the facilities and characteristics of the aquatic environment, specific pools were used for aquatic exercise with dimensions between 8 and 12 m long, 4 and 6 m wide, and 1.20 and 1.65 m deep [18,20]. The water temperature in all interventions was between approximately 30 °C and 33 °C [17,18,19,20].

The duration of the intervention varied in the different articles, with two or three exercise sessions per week (on alternate days), extending the program for 6, 8, 12, or 16 weeks [17,18,19,20]. The effective treatment time of each session was 40 min [19], 45 min [17], 50 min [20], or 60 min [18]. All treatment protocols included a warm-up based on mobility exercises inside or outside the aquatic environment [17,18,19,20], and also included cool-down exercises or a return to calm through floating relaxation [17,18].

One of the studies [17,21] based its treatment on a warm-up with stretching the muscles of the limbs and neck and walking exercises with lateral displacement (15 min), followed by aerobic exercises in three levels (30 min): lower limb exercises sitting on floats; jumping on a trampoline; exercises on an aquatic cycle; and resistance exercises of the upper limbs. It was also the protocol with the highest number of sessions in total [22].

Another of the clinical trials [18] included stretching exercises of the entire posterior and anterior muscle chain through three repetitions of 15 to 25 s and three sets of 15 repetitions for each strength exercise, using shin guards for the lower limbs and floats for the upper extremities. Likewise, another treatment option of aerobic exercises included 6 mobility exercises in the pool versus a total of 12 exercises of mat Pilates focused on trunk control with limb mobility, also using a Pilates ball for relaxation at the end of the session [19]. Otherwise, the last one of the aquatic exercise proposals was associated with an increase in temperature through far-infrared (FIR) therapy versus a placebo method (participants in the intervention group wore t-shirts printed with FIR-emitting ceramic microparticles while subjects in the placebo group wore t-shirts with another composition) [20]. This exercise program included stretching, warm up in water with walking movements, aerobic and strength exercises (walking, running, and jumping, for example: pedaling, kicking the water, relay races, or alternative jumps) and a cool down [20].

In the intervention of Andrade et al. (2019) [17] described in Andrade et al. (2017) [21], exercise control was regulated by heart rate, ventilatory anaerobic threshold, and VO_2_ levels, so that participants went through three levels of aerobic exercise in the main phase of training. The protocol of Salm et al. (2019) [20] measured the intensity of exercise and adapted the sessions using the heart rate, and De Medeiros et al. (2020) [19] moderated the aerobic exercise on the Borg scale.

The effects obtained from the interventions can be considered positive for the state of health and well-being of patients with fibromyalgia [17,18,19,20]. Clinical symptoms, based mainly on pain analysis, improved with aquatic exercise treatment. Specifically, Andrade et al. (2019) [17] indicated a statistically significant reduction in pain on the VAS scale (*p* = 0.05), an impact on quality of life with the FIQ scale (*p* < 0.01), and also an increase in PPT (*p* = 0.05) after treatment. However, after the intervention, this research did not find a statistical correlation between the beneficial effects of the intervention on the clinical manifestations of the disease and changes in markers of exercise intensity and body composition [17]. In addition, in the comparison between groups, the aquatic exercise group presented higher PPT (*p* < 0.01) and well-being (*p* = 0.03) and lower FIQ (*p* < 0.01) and VAS pain (*p* = 0.02) compared to the control group, but no significant differences were observed for the exercise group after the detraining period (*p* > 0.05) in the intergroup analysis.

Therefore, in the difference in means and interaction between groups and follow-up over time, statistically significant differences appeared in favor of the training group compared to the control group. Also, the global score of the SF-36 scale increased in the aquatic treatment groups after the intervention, although the effects on each item will have to be analyzed in depth [17,19].

On the other hand, De Medeiros et al. (2020) [19] showed positive results in reducing pain in both treatment groups, both in an aquatic environment (*p* = 0.001) and with the mat Pilates treatment (*p* = 0.01). Benefits were also found in the scores obtained in the FIQ for both groups (mean difference = 0.91, *p* = 0.002 for the aquatic exercise group; and a mean difference = 1.6, *p* = 0.001 for the mat Pilates group) [19].

Britto et al. (2020) [18] reported a significant reduction in pain by the VAS for both exercise groups, aquatic and land, without appreciable differences in the intergroup comparison. In this study, a statistically significant value was reached in the intragroup mean difference for the aquatic exercise group (*p* < 0.001), changing from a mean of 7.11 ± 2.40 to 5.79 ± 2.62. The reduction of tender points in the aquatic exercise group stands out (*p* = 0.008), and changes were observed in the impact of fibromyalgia on the quality of life via FIQ for the water (*p* = 0.005) group and land group (*p* = 0.006).

Finally, the investigation of Salm et al. (2019) [20] indicated that a combination of temperature increase techniques with aquatic exercise seemed to increase the benefits obtained. Both groups obtained significant changes in pain assessment with the SF-MPQ (*p* < 0.05), and a significant reduction in pain was found in VAS (*p* < 0.0007) with the aquatic exercise and FIR treatment.

## 4. Discussion

Current evidence reflects fibromyalgia as a syndrome characterized by chronic and widespread musculoskeletal pain, accompanied by other medical disorders, which together decrease the quality of life of patients [1,4,13,23]. There are various treatment options, among which the importance of physical exercise stands out [24,25], with aquatic therapy being notable. Water provides an ideal medium for exercise as it allows joint mobilization with reduced effort, presenting low-impact exercises that place less stress on the joints and muscles [26]. Thanks to the various properties of water, various activities and movements can be carried out that are more challenging to perform in dry environments [26,27]. For this reason, aquatic therapy is considered a treatment option to enhance the physical and psychological aspects affected by fibromyalgia.

The analysis of the selected articles supports the implementation of aquatic exercise as a treatment modality for people with fibromyalgia. In general, the results obtained in these investigations showed that it is possible to reduce pain and the impact of fibromyalgia on the quality of life of patients, since statistically significant differences were observed with exercise programs in water and on land [17,18,19,20]. This reinforces the current trend of global monitoring and intervention of patients with fibromyalgia syndrome, so that patients are actively involved in the treatment, and patients have tools to control their symptoms.

The sociodemographic characteristics of the sample were similar in the four studies analyzed [17,18,19,20]; the study population was composed of women from the fourth decade of life, which coincides with the usual age and sex distribution of fibromyalgia [3,10,28]. In addition, there is consensus on using standardized guidelines and established diagnostic criteria for the assessment of fibromyalgia and the inclusion of patients in clinical trials, mainly using the American College of Rheumatology recommendations [10,17,18,19,20,29].

It is also necessary to highlight the high methodological quality of the selected studies; all articles obtained a confirmed score of 8/10 on the PEDro scale [17,18,19,20]. This condition gives the results high reliability and minimizes the risk of bias. The four clinical trials had an adequate randomization process, the follow-up and analysis of results were appropriate, and the initial distribution of the groups was homogeneous, allowing baseline comparability. However, the characteristics of the interventions did not make it possible to blind the patients and therapists; only the article of Salm et al. (2019) [20] blinded the patients, as the aquatic exercise treatment was included in both groups.

Pain (VAS and SF-MPQ) and the impact of fibromyalgia on quality of life (SF-36 or FIQ) were the most studied variables [17,18,19,20]. The assessment of these characteristics becomes relevant in the clinical setting, and this constitutes the priority objective of this review. It allows the results obtained to be transferred to clinical practice. In addition, the inclusion of other variables such as tender points can be highlighted [18], although this assessment is no longer used in the most current guidelines [10,30].

The duration of the interventions (in effective exercise time per session, in total number of sessions, and in weekly distribution) was relatively similar in all analyzed articles, although Salm et al. (2019) [20] concentrated the sessions in 6 weeks and Andrade et al. (2019) [17] opted for 16 weeks of treatment. And only this article conducted a long-term follow-up after a detraining period [17].

Andrade et al. (2019) [17] conducted a comparison between an aquatic exercise group and a control group with inactivity, and it showed benefits in the group that exercised and offered values similar to the baseline when they were subjected to detraining, both in the variables of clinical symptoms and in the control of physical performance. The studies of Britto et al. (2020) [18] and De Medeiros et al. (2020) [19] support the inclusion of exercise in aquatic or land mode. All of this suggests that the symptoms of fibromyalgia could be linked to physical inactivity, with exercise being the basic element of treatment, with low cost and high effectiveness. The effect of exercise could be associated with changes in body composition [31], so Salm et al. (2019) [20] analyzed biochemical markers in the patients. The combination of exercise with changes in body temperature attributable to complementary techniques [32,33] could also modify body composition.

Therefore, aquatic exercise can be considered as a beneficial option for patients with fibromyalgia; the results obtained could be associated with the facilitation of movement in the aquatic environment [34,35]. Performing exercise in water helps improve muscle strength, flexibility, and cardiovascular resistance, within a low-impact environment that allows the intensity to be adapted to the needs of each individual. Furthermore, the changes found could be associated with the patients’ comfort in performing the exercise in an aquatic environment, and the ability to float, considering that it could be more attractive given that many patients experience exercise rejection.

Therapeutic physical exercise plays a fundamental role in the approach of patients with fibromyalgia, being part of the global approach to the pathology, including a program adapted to the needs and preferences of each person within the physical therapy treatment [14,36]. Aquatic exercise can be considered a safe and easy-to-apply exercise modality if the necessary facilities are available. This treatment could reinforce therapeutic adherence, which is especially important [14]. However, the implementation of these exercise programs depends on the availability of pools that allow aquatic exercise and the specialization of the therapists. Sometimes, these circumstances could impair the access of patients to the aquatic exercise modality, being limited by socioeconomic factors. If access to a pool for aquatic exercise is not possible, it is advisable to opt for other forms of exercise that may be equivalent [36].

On the other hand, the comfortable temperature of the water, the adaptation of the exercise to the participant, and individualized supervision could be other important factors to motivate the patient [26,27]. It is also convenient to highlight the effects on general well-being, patient relaxation, and the emotional state, because aquatic treatment is related to a better mood, and the treatments include cool-down exercises with floating maneuvers [18].

Likewise, within a global treatment, aquatic exercise could be related to other therapies with effects on pain modulation, such as transcranial direct current stimulation [37,38,39], or exergame or virtual reality therapies that encourage movement and adherence to exercise [40,41,42]. All of these therapies could be alternated and have a place in the treatment. It would also be advisable to compare the effects associated with each therapy.

The main strength of this review is the analysis of studies with high methodological quality; this makes it possible to translate the results to the healthcare environment. As well, the inclusion of different treatment methodologies based on aquatic exercise, including aerobic and strength exercise, is another strength. On the contrary, certain limitations could be indicated, such as the limited follow-up time in the articles, the lack of blinding of the interventions, the absence of placebo in most studies, or the different aquatic exercise approaches proposed with other associated therapies. Likewise, it should be noted that all studies were carried out on women and on a similar age range. This type of population coincides with the usual fibromyalgia patients, but it could represent a limitation since the generalization of the results could be constrained; for example, the severity of symptoms could be different in chronic pain in both sexes.

Also, the selected search strategy can be considered limited by its simplicity, nevertheless, the search strategy is easily reproducible, so that the results can be replicated and updated in the future by both clinicians and researchers. The intention was that the search formula could offer the most notable results focused on aquatic exercise and fibromyalgia, and that it would be standardized for all databases that were used. Despite selecting a low number of articles, it is necessary to point out that the search was exhaustive and only articles of high methodological quality were selected.

It is advisable to continue conducting future research, in which it could be convenient to carry out a comparative statistical analysis between other treatment therapies with the inclusion of other variables related to physical condition, such as strength levels or electromyographic activity, and use a longer-term follow-up. Based on the studies analyzed as a reference, it is possible to recommend aquatic exercise programs of between 6 and 16 weeks, with training of at least 40 min on alternate days. It seems advisable that the programs be based on aerobic and strength exercise, with mobility of the upper and lower limbs, regulating the intensity through heart rate and the perception of effort on the Borg scale. Future lines of research should delve deeper into these guidelines. At a clinical level, it is advisable to adapt the program to each patient based on general recommendations.

## 5. Conclusions

Aquatic therapy offers physical and psychological benefits in the treatment of fibromyalgia. However, in some instances, the results of this type of approach may not be superior to other exercise options for this pathology. Therefore, it is important to individualize treatment to the needs of patients. Consequently, it is possible to recommend aquatic exercise according to the modalities described in the articles included in this review.

## Figures and Tables

**Table 1:** Characteristics of the trials included in the systematic review.

Author (Year)	Participants (Groups)	Clinical Variables	Assessment Time	Frequency of Treatment
Andrade et al. (2019) [17]	N = 54 (27/27)	VAS FIQ SF-36 BAI BDI PPT PSQI	Baseline Post-treatment 32 weeks	32 sessions in 16 weeks
Britto et al. (2020) [18]	N = 33 (16/17)	VAS FIQ TP Flexibility	Baseline Post-treatment	24 sessions in 8 weeks
De Medeiros et al. (2020) [19]	N = 42 (21/21)	VAS FIQ SF-36 FABQ PRCTS PSQI	Baseline Post-treatment	24 sessions in 12 weeks
Salm et al. (2019) [20]	N = 28 (14/14)	VAS FIQ SF-MPQ	Baseline Post-treatment	18 sessions in 6 weeks

Abbreviations. BAI: Beck’s Anxiety Inventory; BDI: Beck’s Depression Inventory; FABQ: Fear Avoidance Beliefs Questionnaire; FIQ: Fibromyalgia Impact on Quality of Life; PPT: Pressure Pain Threshold; PRCTS: Pain-Related Catastrophizing Thoughts Scale; PSQI: Pittsburgh Sleep Quality Index; SF-36: Short Form-36 Health Survey; SF-MPQ: Short Form McGill Pain Questionnaire; TP: Tender Points; VAS: Visual Analog Scale.

**Table 2:** Interventions and results of the trials that were included in the systematic review.

Author (Year)	Aquatic Exercise Treatment	Alternative Treatment	Results
Andrade et al. (2019) [17]	45′ per session (stretching the muscles of the limbs and neck; walking exercises and lateral displacement; lower limb exercises sitting on floats; jumping on a trampoline; exercises on an aquatic cycle; resistance exercises of upper limbs; floating relaxation).	The control group was instructed to maintain their baseline levels of physical activity	↓ VAS ↓ FIQ ↑ SF-36 ↓ BAI =BDI ↑ PPT =PSQI
Britto et al. (2020) [18]	60′ per session (warm up; active stretching; strengthening exercises for limbs; floating relaxation).	60′ per session (warm-up, active stretching, strengthening, and relaxation equivalent to aquatic exercise)	↓ VAS ↓ FIQ ↓ TP ↑ Flexibility
De Medeiros et al. (2020) [19]	40′ per session (warm up; six mobility exercises of limbs with coordination between flexion of upper and lower limbs; cool down. Intensity exercises moderated by the Borg scale).	50′ per session of mat Pilates (9 main exercises based on core motor control and lower limb mobility, series of 8 repetitions, progression from 1 to 3 series; 3 relaxation exercises, 30′ for each)	↓ VAS ↓ FIQ ↑ SF-36 ↓ FABQ =PRCTS =PSQI
Salm et al. (2019) [20]	50′ per session (stretching exercise; aerobic warm-up; passive stretching; aerobic aquatic fast walking, running, and jumping; strength exercise of limbs and trunk; cool down).	Aquatic therapy in both groups	↓ VAS ↓ FIQ ↓ SF-MPQ

Abbreviations. BAI: Beck’s Anxiety Inventory; BDI: Beck’s Depression Inventory; FABQ: Fear Avoidance Beliefs Questionnaire; FIQ: Fibromyalgia Impact on Quality of Life; PPT: Pressure Pain Threshold; PRCTS: Pain-Related Catastrophizing Thoughts Scale; PSQI: Pittsburgh Sleep Quality Index; SF-36: Short Form-36 Health Survey; SF-MPQ: Short Form McGill Pain Questionnaire; TP: Tender Points; VAS: Visual Analog Scale; ↑: increase; ↓: decrease.

**Table 3:** PEDro score details.

Author (Year)	PEDro Scale											
	1	2	3	4	5	6	7	8	9	10	11	Score
Andrade et al. (2019) [17]	Y	Y	Y	Y	N	N	Y	Y	Y	Y	Y	8/10
Britto et al. (2020) [18]	Y	Y	Y	Y	N	N	Y	Y	Y	Y	Y	8/10
De Medeiros et al. (2020) [19]	Y	Y	Y	Y	N	N	Y	Y	Y	Y	Y	8/10
Salm et al. (2019) [20]	N	Y	N	Y	Y	N	Y	Y	Y	Y	Y	8/10

Abbreviations. N: No; Y: Yes. Details of items. 1: Eligibility criteria; 2: Random allocation; 3: Concealed allocation; 4: Baseline comparability; 5: Blind subjects; 6: Blind therapists; 7: Blind assessors; 8: Adequate follow-up; 9: Intention-to-treat analysis; 10: Between-group comparisons; 11: Point estimates and variability. Note: Eligibility criteria (item 1) does not contribute to total score. Score was checked on PEDro website.

## Data Availability

Data are contained within the article.
